# Free-living bacteria stimulate sugarcane growth traits and edaphic factors along soil depth gradients under contrasting fertilization

**DOI:** 10.1038/s41598-022-25807-w

**Published:** 2023-04-18

**Authors:** Nyumah Fallah, Muhammad Tayyab, Ziqi Yang, Ziqin Pang, Caifang Zhang, Zhaoli Lin, Lahand James Stewart, Mbuya Sylvain Ntambo, Ahmad Yusuf Abubakar, Wenxiong Lin, Hua Zhang

**Affiliations:** 1 Key Laboratory of Sugarcane Biology and Genetic Breeding, Ministry of Agriculture, Fujian Agriculture and Forestry University, Fuzhou, 350002 China; 2 Fujian Provincial Key Laboratory of Agro-Ecological Processing and Safety Monitoring, College of Life Sciences, Fujian Agriculture and Forestry University, Fuzhou, 350002 China; 3 Département de Phytotechnie, Faculté des Sciences Agronominiques, Université de Kolwezi, Kolwezi, Democratic Republic of Congo

**Keywords:** Microbiology, Molecular biology, Plant sciences, Ecology

## Abstract

Free-living bacterial community and abundance have been investigated extensively under different soil management practices. However, little is known about their nitrogen (N) fixation abilities, and how their contributions to N budgets impact plant growth, yield, and carbon (C) and N cycling enzymes in a long-term consecutive sugarcane monoculture farming system, under contrasting amendments, along different soil horizons. Here, *nifH* gene amplicon was used to investigate diazotrophs bacterial community and abundance by leveraging high-throughput sequencing (HTS). Moreover, edaphic factors in three soil depths (0–20, 20–40, and 40–60 cm) under control (CK), organic matter (OM), biochar (BC), and filter mud (FM) amended soils were investigated. Our analysis revealed that β-glucosidase activity, acid phosphatase activity, ammonium (NH_4_^+^-N), nitrate (NO_3_^–^N), total carbon (TC), total nitrogen (TN), and available potassium (AK) were considerably high in 0–20 cm in all the treatments. We also detected a significantly high proportion of Proteobacteria and *Geobacter* in the entire sample, including *Anabaena* and *Enterobacter* in 0–20 cm soil depth under the BC and FM amended soils, which we believed were worthy of promoting edaphic factors and sugarcane traits. This phenomenon was further reinforced by network analysis, where diazotrophs bacteria belonging to Proteobacteria exhibited strong and positive associations soil electrical conductivity (EC), soil organic matter content (SOM) available phosphorus (AP), TN, followed by NH4^+^-N and NO_3_^–^N, a pattern that was further validated by Mantel test and Pearson’s correlation coefficients analyses. Furthermore, some potential N-fixing bacteria, including *Burkholderia*, *Azotobacter*, *Anabaena,* and *Enterobacter* exhibited a strong and positive association with sugarcane agronomic traits, namely, sugarcane stalk, ratoon weight, and chlorophyll content. Taken together, our findings are likely to broaden our understanding of free-living bacteria N-fixation abilities, and how their contributions to key soil nutrients such as N budgets impact plant growth and yield, including C and N cycling enzymes in a long-term consecutive sugarcane monoculture farming system, under contrasting amendments, along different soil horizons.

## Introduction

Globally, sugarcane is one of the main economic crops and is regarded for its high sugar content and bioenergy^1,2^. China is the third-largest sugarcane-producing country worldwide, with Guangxi province accounting for approximately 60% of the total sugar production in China^3^. Sugarcane consecutive monoculture farming system is widely practiced in China due to insufficient land and inadequate judicious planting concepts^4^. However, long-term sugarcane continuous cropping can have deteriorating effects on essential soil nutrients in sugarcane rhizosphere zones as well as induce the proliferation of soil-borne diseases^5^, which may eventually impede the overall productivity of sugarcane^6^. These phenomena have been observed in crops, such as soybeans^7^ and bananas^8^. In a recent study, Pang et al.^6^ demonstrated that continuous sugarcane cultivation had profound negative impacts on sugarcane agronomic parameters, soil fertility, and soil microbial community.

Fertilization is generally carried out to improve crop productivity^9,10^. For instance, sugarcane growers in Guangxi province apply nitrogen (N) fertilizer at the rate of 600–800 kg N ha^−1^, which is 6–8 times more than the average N application rate in Brazil^3^. On the other hand, the utilization of high-dose of N fertilizer in sugarcane continuous cropping fields may not only negatively influence soil fertility and health^11,12^, but may also adversely alter soil microbial and crop growth^13^. Hence, there is mounting pressure on how to safely enhance agricultural productivity. Organic fertilization has an obvious positive effect on soil microbial biomass, functional diversity^14^, and soil enzyme activities^15^ compared with mineral fertilizer^16^. Francioli et al.^17^ reported that bacterial diversity under organic fertilization significantly improved. In our previous study, biochar (BC) amended soil significantly increased the stalk weight and height of sugarcane, improved soil NO_3_^−^, NH_4_^+^, OM, TC, and AK, and had a profound impact on the abundance of diazotrophs genera^18^.

Additionally, environmental concerns and the desire for producing food using an eco-friendly approach^19^ have led farmers to seek more suitable N management strategies^20^. Interestingly, it is worth noting that opting for biological N-fixation (BNF) is an ameliorative strategy because it can provide nutrients for crops^21^, thus boosting crop production capacity^22^, and also maintaining a sustainable terrestrial ecosystem^23^. BNF is the major biological mechanism by which N is available to plants, which is performed by prokaryotic bacteria called diazotrophs^21^. Free-living N-fixing bacteria inhabiting soils contribute considerably to the N budgets of many ecosystems, which are vital for the growth and development of crops. However, soil N cycle has been disturbed unprecedentedly by the excessive use of synthetic fertilizers^24^, thus shifting a diverse range of microbial activities and communities^25^. For instance, Tan et al.^26^ and Berthrong et al.^27^ mentioned that the utilization of N fertilizers diminished diazotrophic community. In a related study, Feng and colleagues pointed out that long-term chemical fertilizer utilization significantly altered soil diazotrophic community structure and led to a decrease in diazotrophs diversity^28^. However, little is known about diazotrophs N-fixation abilities and how their contributions to N budgets impact plant growth and yield, including C and N cycling enzymes in a long-term consecutive sugarcane monoculture farming system, under contrasting amendments, along different soil horizons (0–20, 20–40, and 20–60 cm). To fill these knowledge gaps, we leveraged high-throughput sequencing (HTS) to investigate diazotrophs N-fixation abilities, and how their contributions to N budgets impact plant growth and yield, including C and N cycling enzymes in a long-term consecutive sugarcane monoculture farming system, under contrasting amendments, along different soil horizons.

## Results

### Effects of different fertilization methods on sugarcane agronomic traits

**Figure 1:**
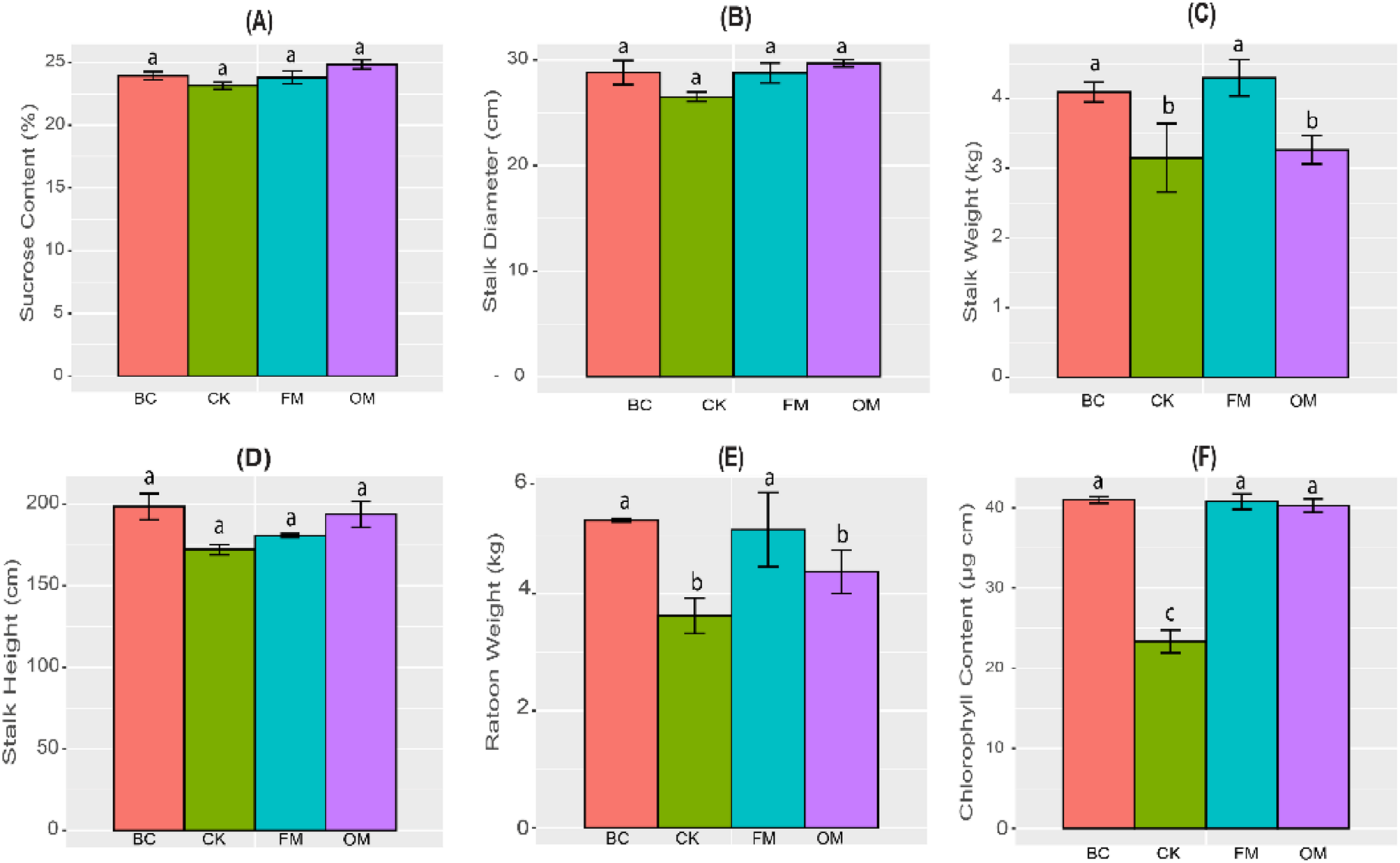
Sugarcane agronomic parameters, including sucrose content (**A**), stem diameter (**B**), stalk weight (**C**), stalk height (**D**), ratoon weight (**E**), and chlorophyll content (**F**) response to biochar (BC), filter mud (FM), organic matter (OM) amended soils, and control (CK).

We noticed that the BC, OM, and FM treatments did not improve the sucrose content, stem diameter, and stalk height compared with the CK treatment (Fig. 1A,B,D). On the other hand, the BC and FM treatments significantly increased (*p* < 0.05) sugarcane stalk weight and ratoon weight compared with the CK and OM treatments (Fig. 1C,E), while chlorophyll content peaked significantly (*p* < 0.05) under the BC, FM, and OM treatments compared with the CK treatment (Fig. 1F).

### Effects of different fertilization methods on edaphic factors

**Figure 2:**
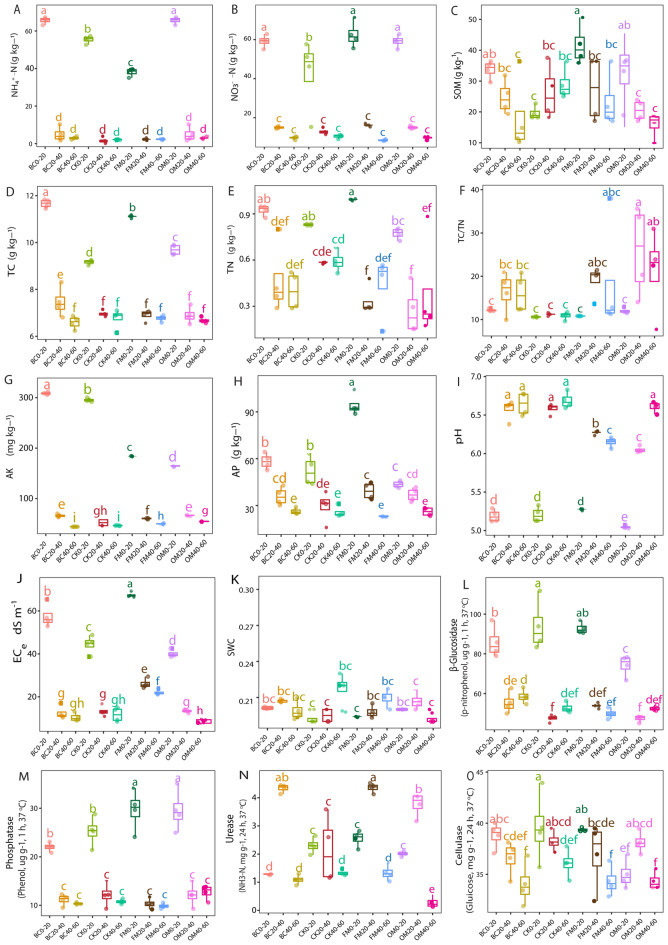
Boxplot illustrating edaphic factors (**A**–**O**) along various soil depths (0–20, 20–40, and 40–60 cm) under the different treatments. Different lowercase letters depict significant differences between treatments (Tukey test, *p* < 0.05).

Here, the BC and OM treatments significantly improved (*p* < 0.05) ammonium (NH_4_^+^N) in 0–20 soil depth compared with the CK treatment. However, the FM treatment significantly decreased (*p* < 0.05) NH_4_^+^-N in 0–20 soil depth compared with the CK treatment (Fig. 2A). Moreover, soil nitrate (NO_3_^–^N) significantly increased (*p* < 0.05) in all the treatments in 0–20 cm soil depth (Fig. 2B) compared with the CK treatment. In 0–20 cm soil depth, soil organic matter content (SOM) significantly increased (*p* < 0.05) under the BC, FM, and OM amended soils compared with the CK treatment. However, SOM was not influenced under the the OM amended soil across 20–40 and 40–60 cm soil depths compared with the CK treatment (Fig. 2C). Furthermore, soil total carbon (TC) was enhanced significantly (*p* < 0.05) under all the amended soils compared with the CK treatment in soil depth 0–20 cm (Fig. 2D). However, soil total nitrogen (TN) and TC/TN were not significantly impacted under the BC, FM, and OM treatments, especially in the first soil depth (0–20 cm) compared with the CK treatment (Fig. 2E,F). Soil available potassium (AK) was significantly higher (*p* < 0.05) in 0–20 and 20–40 cm soil depths compared with the CK treatment, but significantly decreased (*p* < 0.05) in soil depth 0–20 cm under the FM and OM treatments compared with the CK treatment. We also observed that soil AK significantly peaked (*p* < 0.05) under the FM and OM treatments in 20–40 and 40–60 cm soil depths compared with the CK treatment (Fig. 2G). On the other hand, soil available phosphorus (AP) revealed no significant change in the entire soil depth in the BC treatment compared with the CK treatment. Whereas the FM treatment significantly increased (*p* < 0.05) soil AP in soil depths 0–20 and 20–40 cm compared with the CK treatment. While soil AP significantly increased (*p* < 0.05) in 0–20 cm soil depth under the OM treatment, but it was not significantly impacted in the 20–40 and 40–60 cm soil depths compared with the CK (Fig. 2H). Additionally, soil pH was not affected under the BC treatment across the entire soil depth compared with the CK treatment. Moreover, soil pH significantly reduced (*p* < 0.05) under the FM treatment in soil depths 20–40 and 40–60 cm. Whereas soil pH in soil depths 0–20 and 20–40 cm significantly reduced (*p* < 0.05), but was remarkably high (*p* < 0.05 in) in 40–60 cm soil depth under the OM treatment compared with the CK treatment (Fig. 2I). Besides, soil EC increased significantly (*p* < 0.05) under the BC and FM treatments in soil depths 0–20 cm, and 0–20 and 20–40 cm compared with the CK treatment, respectively. While the OM treatment significantly diminished soil EC in soil depth 0–20 cm compared with the CK treatment (Fig. 2J). Whereas soil SWC showed no significant difference under the entire treatment (Fig. 2K).

Meanwhile, β-glucosidase was significantly reduced (*p* < 0.05) in the BC and OM treatments in 0–20 cm soil depth compared with the FM and CK treatments (Fig. 2L). Soil acid phosphatase was considerably improved (*p* < 0.05) in 0–20 cm soil depth under both FM and OM treatments, while the BC amended soil revealed no difference relative to those under the CK treatment (Fig. 2M). Moreover, urease activity under the BC, FM, and OM treatments was significantly higher (*p* < 0.05) in 20–40 cm soil depth compared with the CK treatment (Fig. 2N). In addition, cellulose activity decreased with increasing soil depth under the BC and FM treatments relative to those in the CK treatment. However, cellulose activity significantly reduced (*p* < 0.05) in 0–20 cm soil depth in the OM treatment compared with the CK treatment (Fig. 2O).

### Effects of different fertilization methods on *nifH* gene copies and alpha diversity

Both BC and OM amended soils significantly diminished the *nifH* gene in 0–20 cm soil depth compared with the CK treatment. On the other hand, the FM treatment significantly increased *nifH* gene in 20–40 and 40–60 cm soil depths compared with the CK treatment. Regarding different soil depths, *nifH* gene was stable in the entire soil depth in the BC amended soil, but higher in 0–20 cm soil depth compared with 20–40 and 40–60 cm soil depths in the CK treatment. Furthermore, *nifH* gene was significantly high (*p* < 0.05) in 20–40 cm soil depth compared with 0–20 cm under the FM treatment, but decreased with soil depth in the OM treatment (Fig. S1A). Diazotrophs community diversity was analyzed using diversity estimator (Shannon and Simpson) and richness (Ace and Chao1). The analysis revealed that diazotrophs diversity and richness under various soil amendments exhibited no significant change in the entire soil depth compared with the CK treatment (Table S1).

### Dominant diazotrophs phyla and genera response to different soil amendments

**Figure 3:**
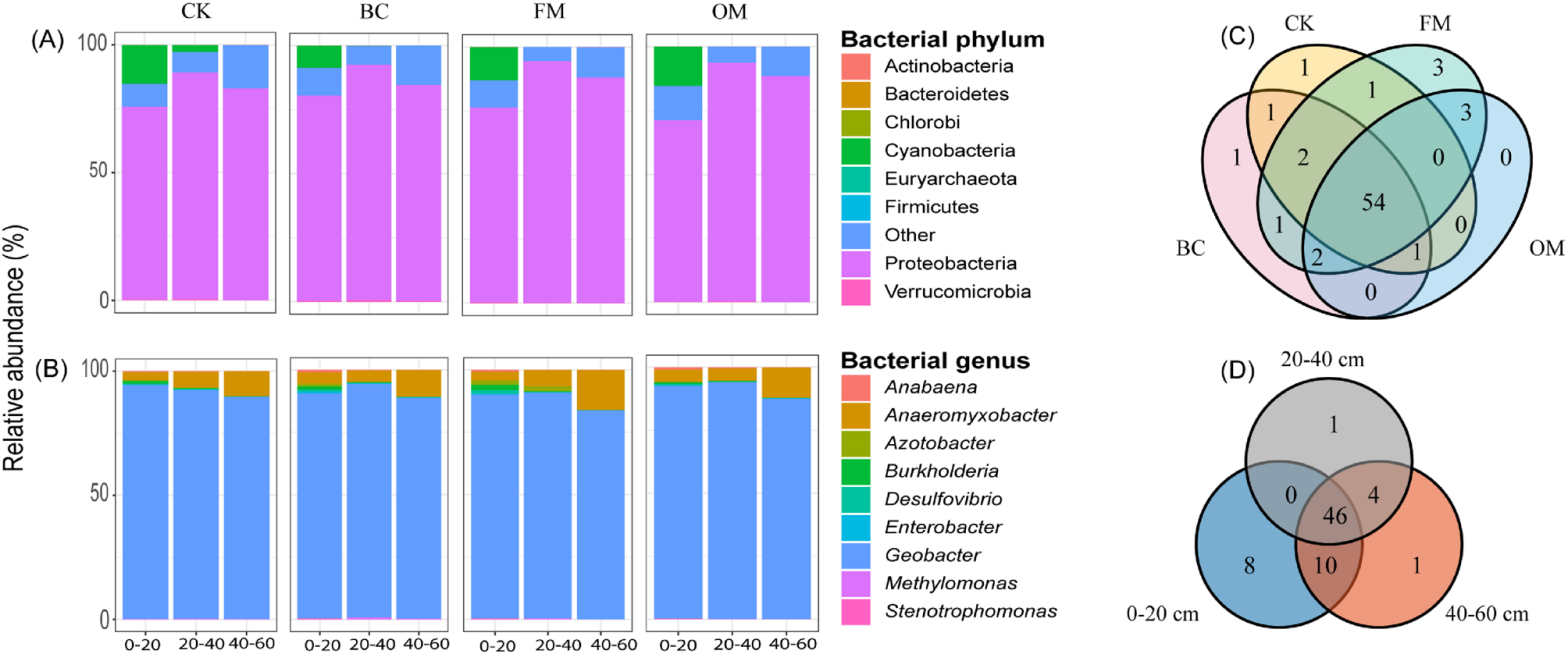
Distribution of diazotrophs phyla (**A**) and genera (**B**), “Other” indicates those identified phyla and genera that were beyond the top nine phyla and genera. Venn diagram illustrating unique and overlap genera under the different treatments  in the various soil depths D.

The dominant diazotrophs relative abundance was examined in the different soil depths (0–20, 20–40, and 40–60 cm) at the phyla and genera levels. We observed that soil depth 0–20 cm was dominantly occupied by diazotrophs phyla, namely, Proteobacteria (71.1–80.2%) and Cyanobacteria (8.6–15.3%). Moreover, 20–40 cm soil depth was characterized by Proteobacteria (88.6–94.4%) and Cyanobacteria (0.0–2.8%), while 40–60 cm was dominated by Proteobacteria (82.9–88.4%) (Fig. 3A). However, the FM, OM, and BC amended soils had little impact on diazotrophs phyla compared with the CK treatment in the entire soil depth (Fig. S1B–I). At diazotrophs genera level, *Geobacter* (89.8–94.3%), *Anaeromyxobacter* (3.2–5.1%), *Burkholderia* (0.8–2.2%), *Azotobacter* (0.1–1.7%), *Desulfovibrio* (0.3–1.5%), *Anabaena* (0.4–1.0%), and *Enterobacter* (0.1–0.5%) were the dominant bacterial genera in 0–20 cm soil depth. Furthermore, *Geobacter* (90.6–94.0%) and *Anaeromyxobacter* (4.7–6.6%) were the dominant genera in soil depth 20–40 cm. In 40–60 cm soil depth, *Geobacter* (83.7–89.5%) and *Anaeromyxobacter* (10.0–16.1%) were abundant (Fig. 3B). Further analysis showed that a vast majority of diazotrophs genera were altered significantly in the different soil depths under the different soil amendments (Fig. S1J–S). Noticeably, *Anabaena* was significantly (*p* < 0.05) higher in 0–20 cm soil depth in the BC amended soil than the other treatments (Fig. S1J). In addition, *Burkholderia, Desulfovibrio*, and *Enterobacter* in soil depth 0–20 cm under the FM and BC treatments performed better compared with the OM and CK treatments (*p* < 0.05) (Fig. S1M–O)*.* Whereas *Methylomonas* in soil depth 20–40 cm peaked significantly (*p* < 0.05) under the BC treatment relative to that under the CK, OM, and FM treatments (Fig. S1Q). However, *Geobacter* diminished in soil depths 0–20 and 40–60 cm, while *Stenotrophomonas* was promoted in 0–20 cm soil depth under the entire treatment (Fig. S1P,S). The unique and overlapping N-fixing genera between the different treatments and soil depths were explored using a Venn diagram. It was observed that 1 genus was unique in both CK and BC treatments, 3 in the FM, and none in the OM amendment (Fig. 3C). Moreover, 8 genera were unique in 0–20 cm and 1 in both 20–40 and 40–60 cm soil depths (Fig. 3D).

### Diazotrophs alpha diversity, *nifH* gene, and edaphic factors response to soil depths and fertilizations

Multivariate ANOVA analysis was leveraged to test the effects of soil depth gradient and fertilization on different soil parameters relating to diazotrophs, namely, OTUs, Shannon, Chao1, coverage, *nifH* gene copies, and edaphic factors, such as urease, cellulase, β-glucosidase and acid phosphatase (Table 1). It was revealed that soil depth significantly (*p* < 0.05) impacted diazotrophs richness index (Chao1) and diversity index (Shannon), followed by diazotrophs coverage. However, soil depth had no impact on *nifH* gene copy number. Furthermore, both soil depth and treatment had a significant impact on bacteria OTUs, while the various treatments had little impact on diazotrophs coverage. Moreover, soil enzyme activities, namely, urease, β-glucosidase, acid phosphatase, followed by cellulase were affected to a greater extent by the different soil depths compared with the different treatments (Table 1). Likewise, edaphic factors, namely, soil pH, AP, AK, TC, TN, NH_4_^+^-N, and NO_3_^–^N were significantly influenced by the different soil depths than the various treatments, while the interaction of the different treatments and soil depths had little impact on soil TC/TN. However, both treatment and soil depth revealed no impact on SOM (Table 2).

**Table 1:** Multivariate ANOVA analysis revealing diazotrophs alpha diversity, *nifH* gene, and edaphic factors response to soil depth and fertilization.

Factor	OTUs	Shannon	Chao1	Coverage	*nifH*	Urease	Cellulase	β-Glucosidase	Acid phosphatase
Treatment	**	NS	NS	*	***	NS	NS	NS	NS
Depth	**	***	***	**	NS	***	***	***	***
T x D	***	***	***	***	***	***	***	***	***

D stands for 0–20 cm, 20–40 cm and 40–60 cm soil depths. T stands for the  CK, BC, FM, and OM treatments. Note: asterisk mark symbolizes the significance level. ***p < 0.001, **p < 0.01, and *p < 0.05.

**Table 2:** Multivariate ANOVA revealing the effects of soil depth and fertilization on edaphic factors.

Factor	pH	EC	SWC	TN	TC	TC/TN	AP	SOM	AK	NO_3_^–^N	NH_4_^+^-N
Treatment	NS	NS	NS	NS	NS	*	NS	NS	NS	NS	NS
Depth	***	***	***	***	***	*	***	NS	***	***	***
T x D	***	***	***	***	***	*	***	**	***	***	***

D stands for 0–20 cm, 20–40 cm and 40–60 cm soil depths. T represents the different fertilizations. Note: asterisk mark symbolizes the significance level. ***p < 0.001, **p < 0.01, and *p < 0.05.

### Diazotrophs community compositions under contrasting fertilizations along different soil depths

**Figure 4:**
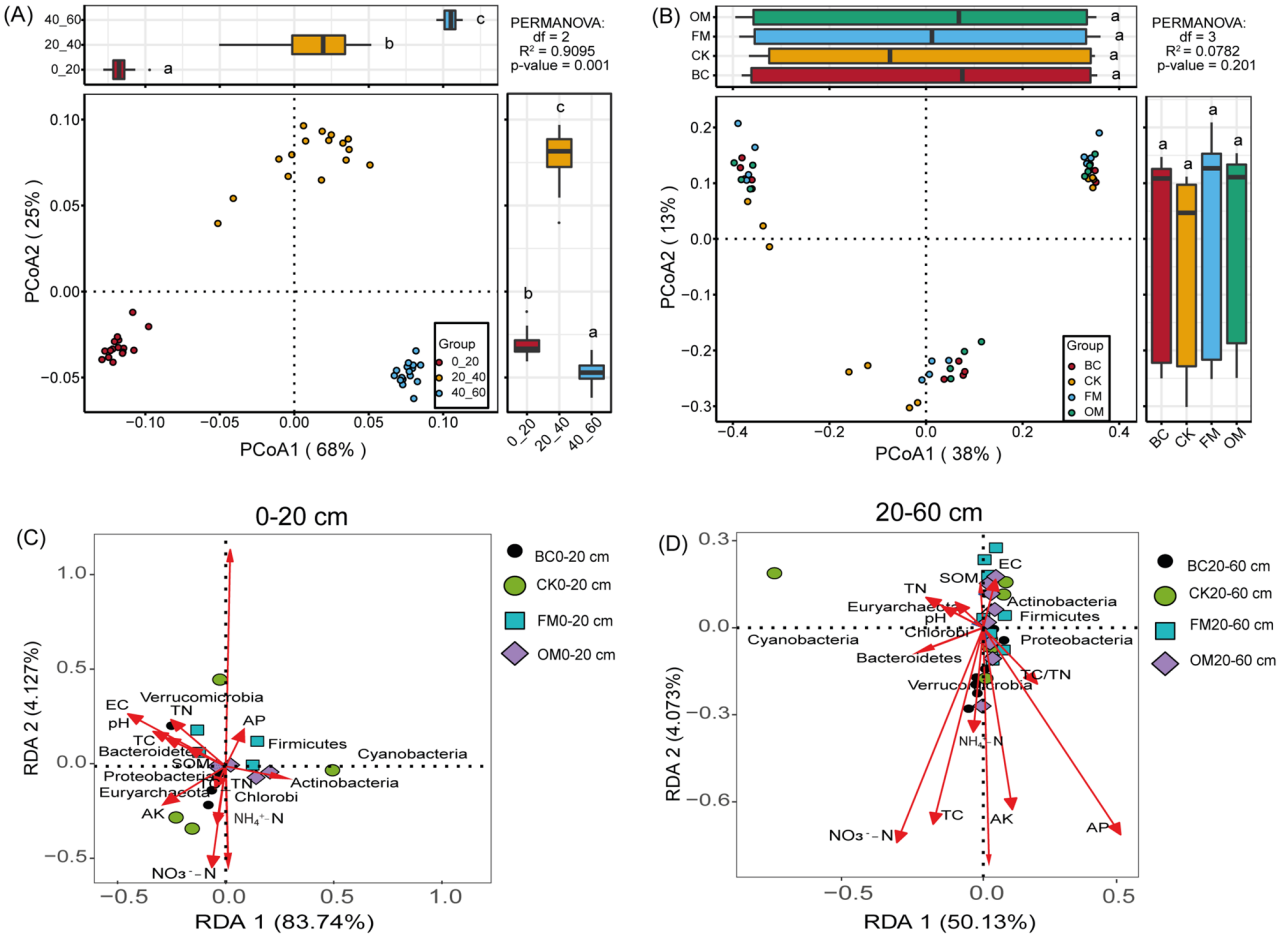
Principal coordinates analysis (PCoA) depicting diazotrophs bacterial community composition in soil depths 0–20 cm and 20–60 cm (**A**,**B**), followed by redundancy analysis (RDA) rivaling the effect of edaphic factors on diazotrophs genera (**C**,**D**) under the different amendments.

Principal coordinates analysis (PCoA) was adopted to assess diazotroph community composition in the different soil depths and the different soil amendments. The analysis demonstrated that diazotrophs community composition in the entire soil depth and the different treatments exhibited distinct distribution patterns (Fig. 4A,B).  Later, redundancy analysis (RDA) was employed separately in two soil depths (0–20 and 20–60 cm) to assess the impact of edaphic factors on diazotrophs community composition at the phyla level. The analysis showed that soil AP (*R*^2^ = 1.1860, *p* < 0.05), EC (*R*^2^ = 1.0933, *p* < 0.05), NH_4_^+^-N (*R*^2^ = 1.0915, *p* < 0.05), TN (*R*^2^ = 1.9840, *p* < 0.05), SOM (*R*^2^ = 1.8575, *p* < 0.05), followed by soil pH (*R*^2^ = 1.5793, *p* < 0.01) and AK (*R*^2^ = 1.5232, *p* < 0.01) had a significant impact on diazotrophs community composition. Whereas soil TC (*R*^2^ = 1.5702, *p* < 0.05) was the minor factor influencing diazotrophs community composition in 0–20 cm soil depth (Fig. 4C). In 20–60 cm soil depth, soil AP (*R*^2^ = 0.4968, *p* < 0.001), AK (*R*^2^ = 0.4273, *p* < 0.001), and NO_3_^–^N (*R*^2^ = 0.7832, *p* < 0.001) were the major impact factors shifting diazotrophs community composition, while TC (*R*^2^ = 0.2532, *p* < 0.01) and EC (*R*^2^ = 0.2184, *p* < 0.01) were the minor drivers altering diazotrophs community composition in 20–60 cm soil depth (Fig. 4D).

### Correlation between edaphic factors and diazotrophs community composition

**Figure 5:**
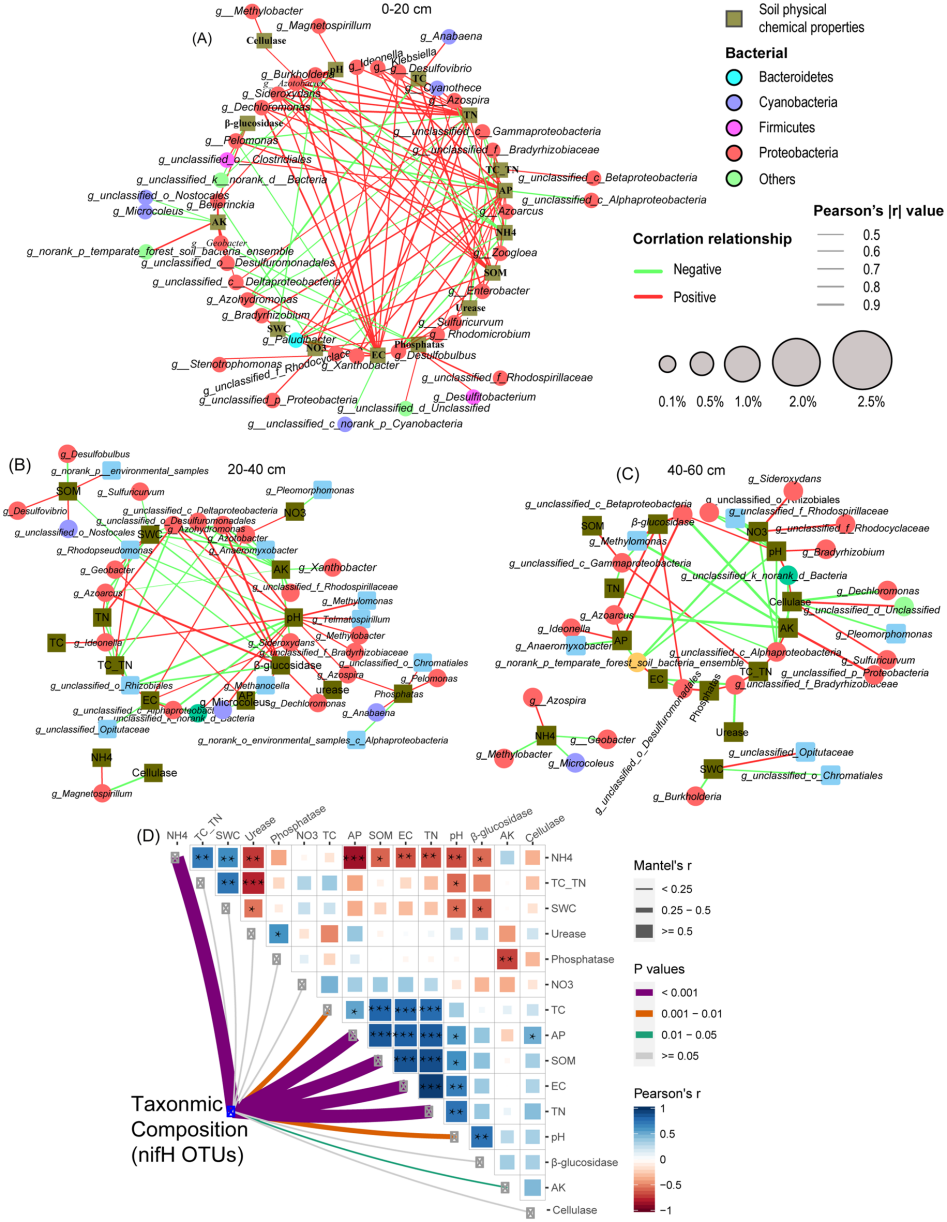
Correlation network analysis depicting the interaction between specific diazotrophs genera and edaphic factors in each soil depth. Red and green lines indicate positive and negative associations, respectively (**A**–**C**). Pairwise comparisons of edaphic factors are shown with a color gradient depicting Pearson’s correlation coefficients. Mantel test depicts the correlation between diazotrophs taxonomic composition (*nifH* OTUs) and edaphic factors. Each edge width correlates with Mantel’s r statistic for the corresponding distance associations (**D**).

Network correlation analysis was used to test the possible interaction between edaphic factors and diazotrophs genera community composition in each soil depth (Fig. 5A–C, Table S2–S5). It was noticed that the total nodes and edges decreased with increasing soil depth, with 0–20 cm recording the highest number of nodes and edges (125 and 58, respectively), followed by 20–40 cm soil depth (76 and 50, respectively) and 40–60 cm soil depth (55 and 43, respectively). Worth noting, diazotrophs association with edaphic factors recorded the highest positive associations (72.8%) and the lowest negative associations (27.2%) in 0–20 cm soil depth. Whereas 20–40 cm and 40–60 cm soil depths accounted for 52.63% and 49.09% positive associations, and 47.37% and 50.91% negative associations, respectively (Fig. 5A–C, Table S2). Moreover, the patterns in network structure demonstrated that diazotrophs genera belonging to Proteobacteria exhibited a significant and positive (*p* < 0.05) association with a vast majority of edaphic factors, especially soil EC, AP, TN, followed by soil SOM in 0–20 cm soil depth (Fig. 5A, Table S3). Similarly, Proteobacteria and Bacteroidetes exhibited a strong and positive (*p* < 0.05) association with soil pH and β-glucosidase in 20–40 cm soil depth (Fig. 5B, Table S4). Whereas diazotrophs genera belonging to Proteobacteria exhibited a strong and positive (*p* < 0.05) correlation with edaphic factors, including β-glucosidase and AP in 40–60 cm (Fig. 5C, Table S5). To have a comprehensive understanding of the relationship between diazotrophs genera edaphic factors, and sugarcane traits, we adopted Mantle test using diazotrophs OTUs. The analysis demonstrated that the taxonomic composition of *nifH* OTUs showed a significant and positive correlation (*p* < 0.05) with a vast majority of the edaphic factors, including SOM, TN, EC, NH_4_^+^-N, followed by soil pH, TC, and AK (Fig. 5D).

**Figure 6:**
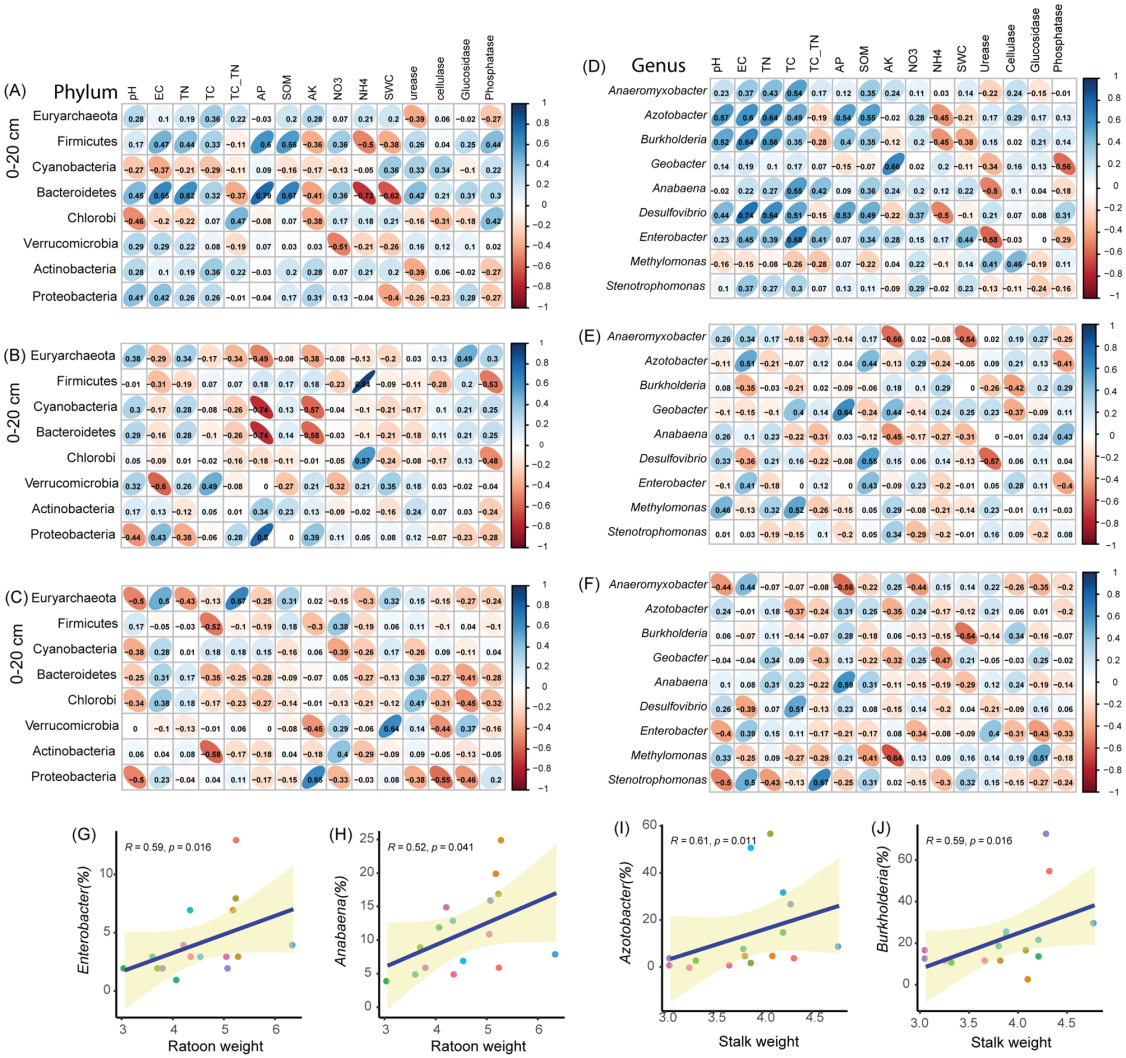
Pearson’s correlation coefficients illuminating the relationships between edaphic factors and the abundant diazotrophs phyla (**A**–**C**); and genera (**D**–**F**) in different soil depths. The heatmap cells marked by “*” or “**” are statistically significant: *p < 0.05 and **p < 0.01. The screened diazotrophs genera were significantly (*p* < 0.001) positively associated with the ratoon and stalk weight of sugarcane (**G**–**J**).

Later, Pearson’s correlation coefficients were employed separately in the various soil depths to further broaden our understanding of how edaphic factors affected the community composition of diazotrophs phyla (Fig. 6A–C) and genera (Fig. 6D–F). It was observed that phylum Bacteroidetes responded strongly and positively (*p* < 0.05) to soil EC, TN, AP, SOM, and NO_3_^–^N. Whereas Firmicutes exhibited a strong and positive association with soil AP and SOM , while phylum Proteobacteria demonstrated a strong and positive (*p* < 0.05) relationship with soil AK in 0–20 cm (Fig. 6A, Table S6). In 20–40 cm soil depth, Proteobacteria was significantly and positively (*p* < 0.05) associated with soil EC, AP, and pH. Whereas Firmicutes had a strong and positive (*p* < 0.05) correlation with soil NH_4_^–^N and acid phosphatase, while phylum Euryarchaeota responded strongly and positively (*p* < 0.05) to β-glucosidase and soil AP. Besides,Cyanobacteria and Verrucomicrobia were significantly and positively (*p* < 0.05) correlated with soil pH and EC, respectively (Fig. 6B, Table S7). In 40–60 cm soil depth, Proteobacteria exhibited a strong and positive (*p* < 0.05) association with cellulose, soil pH, and AK (Fig. 6C, Table S8). Worth noting, majority of diazotrophic genera were significantly and positively (*p* < 0.05) associated with soil edaphic factors compared with diazotrophic phyla, especially in the 0–20 cm soil profile (Fig. 6D–F, Tables S3–S5). To evaluate the association between sugarcane agronomic traits and diazotrophs genera, regression analysis was adopted and suggested that some potential N-fixing bacteria, including *Burkholderia*, *Azotobacter*, *Anabaena,* and *Enterobacter* exhibited a strong and positive (*p* < 0.05) association with sugarcane agronomic traits. For instance, genera such as *Azotobacter* and *Burkholderia* exhibited a strong and positive association with stalk weight (Fig. 6I,J, Table S9), whereas *Enterobacter* had a significant and positive correlation with sugarcane height, ratoon weight, and chlorophyll content (Fig. 6G, Table S9). The analysis also showed that *Anabaena* was significantly and positively associated with sugarcane ratoon weight and chlorophyll content (Fig. 6H, Table S9).

## Discussion

In the current study, we aimed at unraveling diazotrophs N-fixation abilities, and how their contributions to N budgets impact plant growth and yield, including C and N cycling enzymes in a long-term consecutive sugarcane monoculture farming system, under contrasting amendments, along different soil depths. Li et al.^29^ and Orndorff et al.^30^ revealed that organic fertilization enhanced sugarcane growth parameters compared with mineral fertilizers. Similarly, it was observed that the sugarcane stalk and ratoon weight significantly increased under the BC and FM treatments, whereas sugarcane chlorophyll content under various organic amendments peaked significantly compared with the CK treatment. We, therefore, assumed that the accumulations of organic materials in surface soil, which, in turn, could be made available to plants explain the mechanism underpinning the phenomenon ^31^. Furthermore, this result could be ascribed to the large presence of potential N-fixing bacteria detected in the surface soil, as they are known to play significant role in promoting available plant nutrients^32^. Organic fertilization is considered an alternative to inorganic fertilization, with the benefits of enhancing soil nutrients^31,33^. Likewise, we found that edaphic factors such as NO_3_^–^N, TC, and OM contents under the BC, OM, and FM treatments increased significantly, which conformed with previous studies conducted by Yang et al.^34^ and Gopinath et al.^35^. They reported that edaphic factors such as soil N and C accumulation rate, soil pH, and oxidizable organic carbon peaked considerably under organic amendments. We, therefore, hypothesized that the substrate applied may have triggered the proliferation of N-fixation activities, which, in turn, enhanced available soil nutrients. It has also been mentioned that edaphic factors decreased with increasing depths^36,37^. In the current study, soil NH_4_^+^-N, NO_3_^–^N, TC, TN, AK, SOM, and EC were significantly higher in the upper soil depth compared with the subsoil, which is consistent with our previous study^18^, in which soil NH_4_^+^-N, NO_3_^–^N, TC, and TN in the upper soil depth performed better than the subsoil. Soil enzyme activities are considered important indicators of soil fertility due to their pivotal role in soil biochemical reactions, and the maintenance and sustenance of soil fertility and health^38^. Akhtar et al.^39^ and Zhao et al.^40^ documented that OM amended soils increased soil enzyme activity in topsoil and tends to decrease with increasing soil depth^18,41^. Similarly, we observed that β-glucosidase and acid phosphatase were significantly higher in 0–20 cm soil depth in all treatments compared with the subsoil, which may, in part, suggest that surface soil could show a more significant improvement in β-glucosidase and acid phosphatase turnover than subsoil. The increase in these soil enzyme activities in the topsoil in all treatments could be associated with the different soil amendments used^42^.

Environmental gradients such as soil management practices and soil depths are major factors influencing the density of soil microorganisms^36,43^. For instance, Seuradge et al.^44^ reported that soil depth was the primary environmental gradient that affected the bacterial community. In a related study, it was revealed that bacterial abundance was profoundly altered in different soil horizons under straw retention farming systems^41^. Likewise, a vast majority of diazotrophs genera under the various treatments were considerably altered. However, Proteobacteria accounted for a substantial number of bacterial phyla, especially in soil depths 0–20 and 20–40 cm. Proteobacteria are Gram-negative, with outer membranes largely consisting of lipopolysaccharides, and are widely known as a plant growth promoters^45^. Although not many studies have lined Proteobacteria to N-fixation activities as compared with cyanobacterial populations, evidence has emerged that they are worthy of fixing N. For instance, Delmont and his co-workers documented the first genomic evidence for non-cyanobacterial diazotrophs bacteria harboring the surface of ocean waters belonging to Proteobacteria with N-fixing potential. They also mentioned that the detected diazotrophs were remarkably abundant and widespread in both the Atlantic Ocean and the Pacific Ocean^46^, which partly agreed with our finding. We, therefore, inferred that the significantly proportion of Proteobacteria detected in the soil may have played vital role in promoting soil nutrients such as TN, NH_4_^+^-N, and NO_3_^–^N, which, in turn, precipitated the crop traits. Additionally, *Geobacter* bacteria accounted for a substantial portion of the total bacterial genera in the entire soil depth, which is roughly consonant with previous reports documented by Liu et al.^47^ and Liao et al.^48^, in which it was established that *Geobacter* was one of the abundant soil microbial detected in the soil amended with BC. Moreover, recent discoveries have pointed out that *Geobacter* is a newly identified N-fixing bacteria dominant in paddy soils. For example, Masuda et al.^49^ demonstrated that soil N-fixing activity peaked significantly after adding ferrihydrite and ferric iron oxides to the soil, which was primarily driven by *Geobacter* and *Anaeromyxobacter*. In a related study, it was reported that *G. sulfurreducens* was capable of fixing N, which was contingent upon anode respiration^50^, evident by the increase in TN, NH_4_^+^-N, and NO_3_^–^N. Genera *Anabaena* and *Enterobacter* were significantly enhanced in the 0–20 cm in the BC and FM treatments. Studies have revealed that *Anabaena* is capable of fixing N, and it is a filamentous cyanobacteria genera^51,52^. Our findings corroborated with the study conducted by Chen et al.^53^, wherein it was reported that the utilization of BC improved soil microbial abundance in 0–15 cm soil depth. The significant amount of *Anabaena* detected in the surface soil (0–20 cm) may have led to the increase in soil N-related nutrients such as NH_4_^+^-N, NO_3_^–^N, and TN. *Enterobacter* is widely spread in the environment, including soil, plant, water^54^, vegetation, and human feces^55^, and is considered a nosocomial pathogenic bacteria^56^ and a plant growth promoter^57^. For instance, Ji and his colleagues documented that *Enterobacter cloacae* HG-1 strain isolated from saline-alkali soil contained high N-fixation activity and produced plant hormones, iron carriers, and 1-aminocyclopropane-1-carboxylic acid deaminase. They also established that the inoculation of this strain was worthy of enhancing crop agronomic traits, including plant height, root length, dry weight, and fresh weight by 18.83%, 19.15%, 17.96%, and 16.67%, respectively^58^. We, therefore, theorized that the increase in *Enterobacter* in the 0–20 cm under the BC and FM treatments may have led to the increase in NH_4_^+^-N and NO_3_^–^N, which, in turn, could be used by sugarcane plants, thus triggering the growth of sugarcane traits^59,60^.

Soil microbial communities have been reported to be very responsive to soil environmental variables^61,62^. In a study conducted by Pang and his co-workers, it was reported that a vast majority of edaphic factors exhibited a strong and positive regularity effect on bacterial community composition. For instance, some potential N-fixing bacteria such as Bacteroidetes and Verrucomicrobia exhibited a strong and positive correlation with AN, AK, and OM, while Actinobacteria, and Proteobacteria and Cyanobacteria revealed a significant and positive relationship with soil AN and AK, respectively^63^. Similarly, Lian et al. and his colleagues established that soil organic carbon, NH_4_^+^-N, NO_3_^–^N, dissolved organic carbon, and soil pH were the principal factors influencing rhizosphere bacterial dissimilarities under sugarcane-soybean intercropping. Here, RDA analysis showed that soil AP, EC, NH_4_^+^-N, TN, and OM were the major impact factors shifting diazotrophs genera community composition, particularly in 0–20 cm soil depth. This phenomenon was further validated by network analysis, where diazotrophs bacteria belonging to Proteobacteria demonstrated a significant and positive association with soil EC, AP, TN, followed by SOM, especially in the 0–20 cm soil depth. These results were reinforced by Mantle test and Pearson’s correlation coefficients analyses, which is in agreement with Pang et al.^10^ findings, wherein it was pointed out that nitrifying flora and N-fixing flora were significantly associated with soil NO_3_^–^N, pH, and C/N.

A number of studies have investigated plant-microbiome interactions in a quest to identify plant growth-promoting strains, with the aim of promoting more eco-friendly agriculture activities^64^. For example, Kifle et al.^65^ established that the utilization of diazotrophs bacteria strains significantly increased the germination rate of maize seed, root length, seed vigor index, leaf chlorophyll, and dry weight. Correspondingly, we observed that some potential N-fixing bacteria, including *Burkholderia*, *Azotobacter*, *Anabaena,* and *Enterobacter* exhibited a strong and positive (*p* < 0.05) association with sugarcane agronomic traits, namely, sugarcane biomass and ratoon weight, respectively. We, therefore, postulated that this phenomenon was responsible for the marked increase observed in the sugarcane stalk weight, ratoon weight, and chlorophyll content.

## Conclusion

Our study demonstrated that organic soil amendments such as BC, FM, and OM treatments are worthy of enhancing crop agronomic traits as well as edaphic factors, including β-glucosidase, acid phosphatase, NH_4_^+^-N, NO_3_^–^N, OM, TN, and TC, especially in the first soil depth (0–20 cm). Moreover, our findings suggested that the abundance of Proteobacteria, *Geobacter*, *Anabaena*, *Enterobacter*, and *Desulfovibrio* were worth of promoting crop growth traits as well as vital nutrients, including TN, NH_4_^+^-N, NO_3_^–^N, OM, and TC, particularly in the upper soil depth (0–20 cm), evident by the strong and positive association detected between diazotrophs bacteria and edaphic factors. Taken together, our findings are likely to further enhance our understanding of diazotrophs N fixation abilities, and how their contributions to key soil nutrients such as N impact plant growth and yield, including C and N cycling enzymes in a long-term consecutive sugarcane monoculture farming system, under contrasting amendments, along different soil horizons.

## Materials and methods

This study was conducted from March 2018 to December 2020 at the Fujian Agriculture and Forestry University, Sugarcane Research Center, Fuzhou, Fujian Province, China (26°05′00.0″N, 119°13′47.0″E). The site has a clay loam texture soil, with an annual temperature of 20 °C and rainfall of 1369 mm annually. The experiment was laid in a randomized block design consisting of four treatments replicated thrice. The treatments include: control (CK), organic matter (OM), biochar (BC), and filter mud (FM). The size of experimental site was measured 100 m^2^ (25 m × 4 m), with each replicate covering an area of 25 m^2^ (5 m × 5 m). On March 20, 2018, the BC was applied at the rate of 30 t ha^−1^, organic matter at 25.5 t ha^−1^, and filter mud was applied at the rate of 20.5 t ha^−1^. The FM and BC utilized during the study were purchased from Nanjing Qinfeng Crop Straw Technology Company, China. The BC was produced from sugarcane straw at the 550–650 °C and the OM used during the research was composed of pig manure, while FM was obtained from precipitated impurities found in the sugarcane juice that is removed when sugarcane is being processed through filtration, as mentioned by Orndorff et al.^30^ and Elsayed et al.^66^. The basic soil properties were measured before the application of various amendments (Table S10). The different soil amendments were surface applied and immediately mixed into the ploughed soil at the depth of 0–30 cm using rotary tillage before cultivating the sugarcane. Sugarcane stalks were cut at about 10–15 cm in length, with two buds on each sett^67^. Fifteen setts were planted on each row, consisting of 0.3 m between plant-to-plant spacing and 0.5 m row-to-row spacing.

### Soil sampling

Surface soil (0–20 cm), subsoil (20–40 cm), and dipper soil depth (40–60 cm) were sampled in December 2020. Sampling was conducted at five different spots in each plot, homogenized, and mixed accordingly^43^. A portion of each soil sample was air-dried, grounded, and sieved through 2 mm mesh. Sieved soil (2 mm) was used to analyze soil enzyme activities, while the other portion was stored at − 20 °C for the extraction of DNA, ammonium (NH_4_^+^-N), and nitrate (NO_3_^–^N).

### Assessment of sugarcane agronomic traits

Sugarcane heights were determined in centimeters (cm) using a meter rod from the soil surface to sugarcane’s top. The mean of sugarcane heights were determined using the average of three replicates. We used Legendre^68^ approach by milling and measuring the juice for pol and Brox using thirty sugarcane stalks that were randomly sampled from each row. The individual weight of each sugarcane stalk (kg stalk^−1^) was measured using sugarcane plant fresh weighs. Plants were harvested in December 2020, and yield parameters were estimated. A portable chlorophyll meter was used to record the chlorophyll content of ten mature and healthy leaf close to the top in each plot. All the methods we adopted in this study were performed according to relevant rules and guidelines.

### Measurement of edaphic factors under contrasting amendments

Soil edaphic factors, namely, total nitrogen (TN), total carbon (TC), total phosphorus (AP), and available potassium (AK) were determined as mentioned by Bao^69^. A glass electrode pH meter was used for the estimation of soil pH. Fresh soil sample was used to extract soil NH_4_^+^-N and NO_3_^–^N with 2.0 M KCl and measured using the continuous flow analyzer (San++, Skalar, Holland)^70^. Soil OM was assessed by adopting the Walkley − Black approach, which contained the soil OM oxidation by H_2_SO_4_ and K_2_Cr_2_O_7_, and FeSO_4_ was later used for titration^71^. Soil electrical conductivity (EC) was calculated in a 1/5 (w/v) aqueous solution using conductivimeter (Crison mod. 2001, Barcelona, Spain). Soil water content (SWC) was estimated gravimetrically by drying the soil samples in an oven at 105 °C for 12 h and the dried soil samples were later weighed^72^.

The estimation of soil enzyme activities were carried out following the methods reported by Tayyab^73^ and Sun et al.^74^. In brief, cellulose (glucose, mg/g 24 h, 37 °C) was estimated colorimetrically by calculating a decrease in 3,5-dinitrosalicylic acid from reducing sugar using buffer sodium carboxymethylcellulose solution after the soil was incubated. Acid phosphatase activity was measured using a nitrophenyl phosphate disodium substrate (phenol, ug/g, 1 h, 37 °C). β-glucosidase activity was assessed using a colorimetric p-nitrophenol assay after buffering the soil with p-nitrophenyl-β-glucopyranoside, (p-nitrophenyl, μg/g, 1 h, 37 °C). Kandeler and Gerber buffered method was employed to measure soil urea activity by using urea as a substrate.

### Soil DNA extraction

Genomic DNA from all the samples was extracted using the Fast DNA TM Spin kit according to the manufacturer's guideline (MP Biomedical, Santa Ana, CA, USA) which is designed for soil DNA isolation. DNA purification was performed using DNA purification kits according to manufacturer instructions (Tiangen Biotech Co., Ltd., Beijing, China). Nanodrop spectrophotometer was adopted to measure the DNA quality and stored at − 20 °C for further analysis.

### Quantitative real-time PCR assay

Real-time quantitative PCR was used to determine the abundance of the *nifH* gene using MIQE (Minimum Information for Population of qPCR Experiments). The qPCR experiment was performed using SYBR Premix Ex TaxTM (Perfect Real Time) kit with a 7500 Fast Real-Time PCR system. The reaction was carried out in a 25 µL volume containing 12.5 µL of SYBR Premix Ex TaqTM (2 ×, TaKaRa Biotechnology Co.), 0.5 µL ROX Reference dye II (50 ×, TaKaRa Biotechnology Co.), 10 µL dd H_2_O, 1 µL (10–30 ng) DNA template and 0.5 µL (5 µM) using primer set PolF and PolR^75^. *nifH* gene PCR protocols consisted of an initial activation step of 95 °C for 30 s, followed by 40 cycles of 95 °C for 5 s and 34 s at 60 °C. Fragments of the *nifH* gene were cloned into the pMD19-T plasmid and the correct inserted genes were chosen. The potential PCR inhibitors of the DNA samples were determined using serial dilutions. Major inhibitions were not observed in the DNA samples extracted. To develop the standard curve, serially diluting plasmid was used to the final concentrations of 10^8^–10^2^ gene copies number µL^−1^. The qPCR efficiencies were 98% for *nifH* and the R^2^ of the standard was higher than 0.99.

### *nifH* gene sequencing

We conducted high throughput sequencing to investigate diazotrophs community composition using the Illumina Miseq platform. *nifH* gene amplification was conducted using primer pair PolF and PolR^75^ and merged with barcode sequences and Illumina adaptor sequences^76^. Sample libraries were obtained from the products of the purified PCR. We used the Miseq 300 cycle Kit to conduct paired-end sequencing using a Miseq benchtop sequencer (Illumina, San Diego, CA, United States). We separated raw *nifH* gene sequences using sample based on their barcodes and permitting up to one mismatch and carried out quality trimming using Btrim^77^. FLASH was leveraged to merge the forward and reverse reads into full-length sequences^78^, and sequences with short bases were eliminated. We randomly conducted resampling with 10,000 sequences/sample. UCLUST was adopted to categorize the operational taxonomic units (OTUs) at 97% similarity level, and singletons were removed. The frameshift caused by insertions and deletion in DNA sequences were checked and corrected by RDP FrameBox. Later, we translated valid *nifH* gene sequences (300–320 bp) to proteins sequences and taxonomic assignment was carried out using RDP FrameBox tool^79^. Finally, the raw data were submitted to the NCBI Sequence Read Archive (accession no. PRJNA815949).

### Statistical analysis

We examined the differences in mean values between treatments and soil depth using two-way analysis of variance (ANOVA), followed by Tukey's comparison at a 5% significance level^43^. Venn diagram was employed to visualize unique and overlapped diazotrophs genera in the various treatments and soil depths (http://bioinfogp.cnb.csic.es/tools/venny/index.html). The effect of soil depth gradient and fertilization regime on different soil parameters relating to diazotrophs and edaphic factors were tested using multivariate ANOVA in 0–20 cm, 20–40 cm, and 40–60 cm soil depths. Principal coordinate analysis (PCoA) and an analysis of similarities (ANOSIM) were conducted to test if there was a significant difference in diazotrophs community composition in the different soil depths and treatments. We also tested the association between diazotrophs community composition and edaphic factors by adopting redundancy analysis (RDA) in 0–20 and 20–60 cm. The patterns in the network structure of diazotrophs community composition and PERMANOVA (with permutations = 999) analyses were tested using vegan R-package and later generated using ggplot^80^. Mantel test was adopted to examine the relationship between diazotrophs taxonomic composition and edaphic factors using “vegan” package^61^. The correlation between diazotrophs community composition and edaphic factors in the different soil depths was further conducted using diazotrophs genera and phyla by adopting “corrplot” package in R-software^81^, and the significant level was tested using “psych” package. Regression analysis was leveraged to test the relationships between important diazotrophs genera and sugarcane traits using “ggpmisc” package.

## Supplementary Information

Supplementary Figure S1.

Supplementary Tables.

## Data Availability

All Illumina sequence data from the current study are available from the Sequence Read Archive (SRA) of NCBI (National Center of Biotechnology Information) under the BioProject ID PRJNA815949.
