# Supplementary material for: Free-living bacteria stimulate sugarcane growth traits and edaphic factors along soil depth gradients under contrasting fertilization
Source: Sci Rep. 2023 Apr 18;13:6288. doi: 10.1038/s41598-022-25807-w (PMC10113235; doi:10.1038/s41598-022-25807-w)
Supplement: Supplementary file 1 — Supplementary Figure S1. [file 41598_2022_25807_MOESM1_ESM.docx]

**Supplementary material**


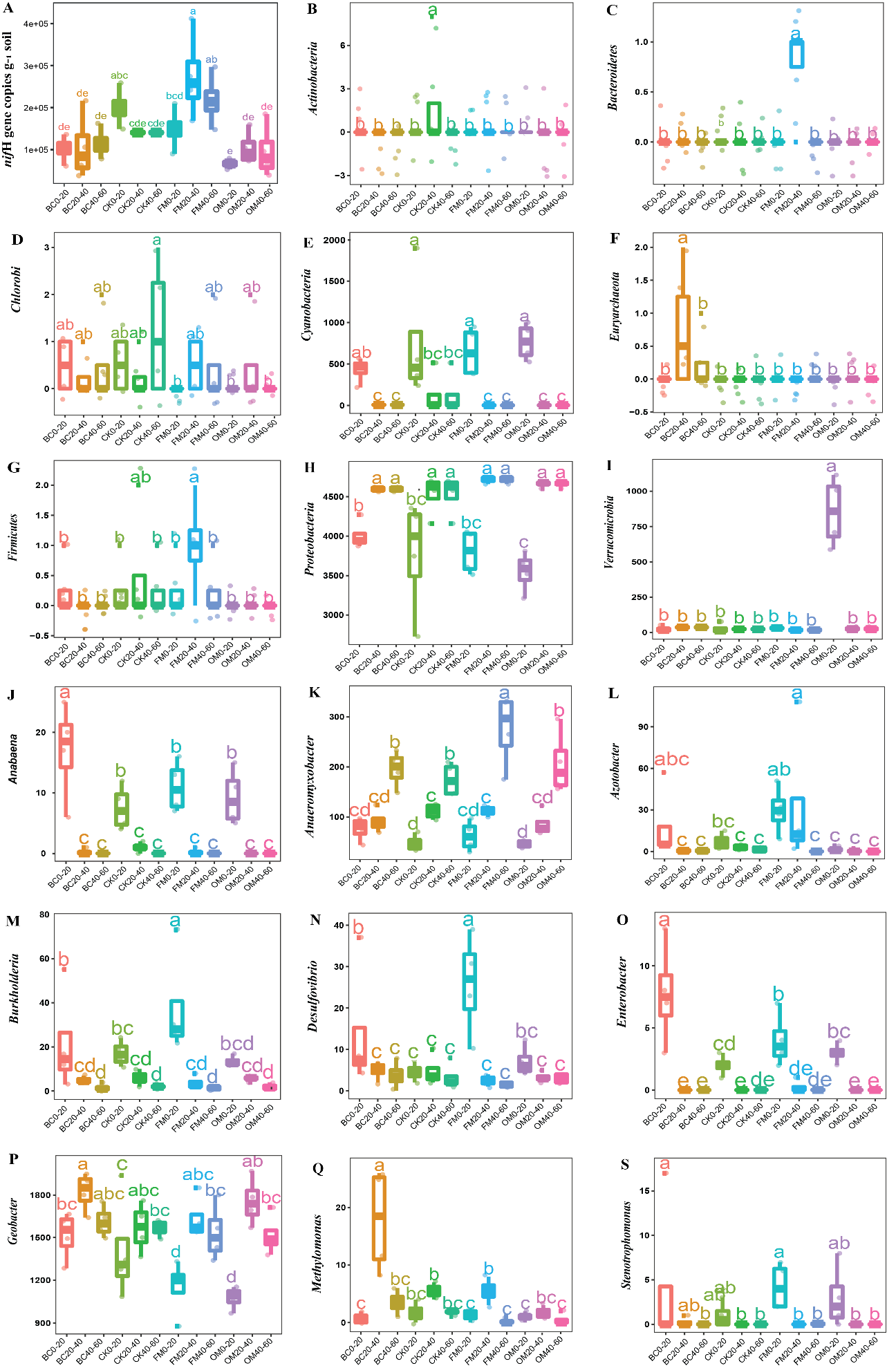


**Figure S1.** Variation in nifH gene copies number (A), dominant N2 fixers phyla (B-I) and genera (K-S) in 0–20 cm, 20–40 cm and 40-60 cm soil layers under BC, biochar amendment; FM, filter mud; OM, organic matter; compared to the CK, inorganic fertilize during sugarcane growing seasons. Boxplots with various lowercase letters indicate a significant difference between treatments based on Tukey’s HSD test (p < 0.05).
